# BMC Gastroenterology reviewer acknowledgment 2015

**DOI:** 10.1186/s12876-016-0430-7

**Published:** 2016-02-19

**Authors:** Elaine Zhang

**Affiliations:** BMC Gastroenterology, BioMed Central, 233 Spring Street, New York, NY 10013 USA

## Abstract

The editors of BMC Gastroenterology would like to thank all our reviewers who have contributed to the journal in Volume 15 (2015).

**Noorizan Abdul Majid**

Malaysia

**Thomas Abrams**

USA

**Florence Abravanel**

France

**Syeda Afroze**

USA

**Daniel Agardh**

Sweden

**Rakesh Aggarwal**

India

**Ferdinando Agresta**

Italy

**Sinead Aherne**

Ireland

**Jawad Ahmad**

USA

**Y Akutsu**

Japan

**Badr Al Bawardy**

USA

**J. Wesley Alexander**

USA

**Alicia Algaba**

Spain

**Turki Alharbi**

Saudi Arabia

**Marco Allaix**

Italy

**Lee-Ann H. Allen**

USA

**Gianfranco Alpini**

USA

**Mary Anne Amalaradjou**

USA

**Kamal Amin**

Saudi Arabia

**Leif Percival Andersen**

Denmark

**Tiing Leong Ang**

Singapore

**Harry Antoniades**

UK

**Toru Aoyama**

Japan

**Christina Arnold**

USA

**Zubin Arora**

USA

**Serhat Avcu**

Turkey

**Matias Avila**

Spain

**Hasan Aydin**

Turkey

**A B**

Germany

**Hideo Baba**

Japan

**Oliver Bachmann**

Germany

**Haibo Bai**

USA

**Michael Bailey**

USA

**Gian Luca Baiocchi**

Italy

**Snigdha Banerjee**

USA

**Claudia Banzato**

Italy

**Edward Barnes**

USA Minor Outlying Islands

**Anushka Baruah**

USA

**Mohammad Bashashati**

Canada

**Guido Basilisco**

Jamaica

**Fateh Bazerbachi**

USA

**Paul Beavis**

Australia

**Anu Behari**

India

**Ajay Belgaumkar**

UK

**Alberto Bellan**

Italy

**Alexander Berezin**

Ukraine

**Marie-Luise Berres**

Germany

**Govind Bhagat**

USA

**Kalyan Bhamidimarri**

USA

**Bhushan Bhole**

India

**Federico Biagi**

Italy

**Itxarone Bilbao**

Spain

**Louise Bill**

Denmark

**Camilla Böckelman**

Finland

**Bruno Bonaz**

France

**Guillaume Bouguen**

France

**Alex Boussioutas**

Australia

**Christopher Bowlus**

USA

**Charlène Brochard**

France

**Christine Budke**

USA

**Elisabetta Bugianesi**

Italy

**Jason Burkhead**

USA

**Nick Burr**

UK

**Carlo Caffarelli**

Italy

**Diego F Calvisi**

Germany

**Fabio Campanile**

Italy

**Jordi Camps**

Spain

**Yin Cao**

USA

**Rocco Cappellesso**

Italy

**Marco Carbone**

Italy

**Andrea Cariati**

Italy

**Bertrand Cariou**

France

**Siobhán Cashman**

UK

**Patricia Castelucci**

Brazil

**Phillipo Chalya**

Tanzania, United Republic Of

**Heyson Chan**

Hong Kong

**Michael Huw Chapman**

UK

**Hrushikesh Chaudhari**

India

**Chieh-Chang Chen**

Taiwan

**Jun Chen**

China

**Ming-Jen Chen**

Taiwan

**Wen-Chi Chen**

Taiwan

**Hsiu-Chi Cheng**

Taiwan

**Giuseppe Chiarioni**

Italy

**Thirunavukkarasu Chinnasamy**

India

**Sakkarin Chirapongsathorn**

Thailand

**King-Wah Chiu**

Taiwan

**A Cho**

Japan

**Evangelos Cholongitas**

Greece

**Bruno Christ**

Germany

**Yu Jo Chua**

Australia

**Seng-Kee Chuah**

Taiwan

**Chen Shuan Chung**

Taiwan

**Michele Cicala**

Italy

**Rachele Ciccocioppo**

Italy

**Sandra Ciesek**

Germany

**Marilisa Citton**

Italy

**Mauro Cives**

Italy

**Marguerite Clyne**

Ireland

**Irene Coati**

Italy

**Mehmet Coban**

Turkey

**Gareth Corbett**

UK

**Francesca Cordero**

Italy

**Maura Corsetti**

Belgium

**Chris Corton**

USA

**Luís Costa Matos**

Portugal

**Melinda Coughlan**

Australia

**Thorsten Cramer**

Germany

**Cesare Cremon**

Italy

**Javier Crespo**

Spain

**Tim Cross**

UK

**Alessandro Cucchetti**

Italy

**Adrian Cummins**

Australia

**Rosario Cuomo**

Italy

**Douglas Curran-Everett**

USA

**Srinivasan Dasarathy**

USA

**Ilva Daugule**

Latvia

**Eric Davies**

USA

**Andrew Day**

Australia

**Paola De Angelis**

Italy

**Nicola De Bortoli**

Italy

**Roberto De Franchis**

Italy

**Richarda De Voer**

Netherlands

**Evelien Dekker**

Netherlands

**Jean Charles Delchier**

France

**Christine Delporte**

Belgium

**Renumathy Dhanasekaran**

USA

**Puneet Dhar**

India

**John Dillon**

UK

**Phil Dinning**

Australia

**Enric Domingo**

UK

**Quanjiang Dong**

China

**Gabriella D'Orazi**

Italy

**Richard Douard**

France

**Jacqueline Doyle**

UK

**Christos Dr Zouboulis**

Germany

**Ignat Drozdov**

UK

**Hanna Dückers-Horst**

Germany

**Philippe Ducrotte**

France

**Pradeep Dudeja**

USA

**Dan Dumitrascu**

Romania

**Cuong Duong**

Australia

**Lynn Dustin**

UK

**Adam Dziki**

Poland

**Sanna Edelman**

Finland

**Piotr Eder**

Poland

**Robert Eferl**

Austria

**Laurent Ehrlich**

USA

**Nikos Eleftheriadis**

Greece

**Abdul Elkadri**

Canada

**Pierre Ellul**

Malta

**Magdy El-Salhy**

Norway

**Christoph Elsing**

Germany

**Cathy Eng**

USA

**Arzu Ensari**

Turkey

**Meira Epplein**

USA

**Askin Erdogan**

Turkey

**Elsa Fabbretti**

Slovenia

**Jan Fallingborg**

Denmark

**Klaudia Farkas**

Hungary

**Per G Farup**

Norway

**Peter Ferenci**

Austria

**Conrado M Fernandez Rodriguez**

Spain

**Jose Fernandez-Cebrian**

Spain

**Christian Fingas**

Germany

**Gionata Fiorino**

Italy

**Gabor Firneisz**

Hungary

**Wolfgang Fischer**

Germany

**P. Marco Fisichella**

USA

**Reidar Fossmark**

Norway

**Giampiero Francica**

Italy

**Martin Freeman**

USA

**Michael Freemark**

USA

**Shunjun Fu**

China

**Ho Fuang**

Malaysia

**Shiki Fujino**

Japan

**David Fuks**

France

**Mathurin Fumery**

France

**Akihiro Funakoshi**

Japan

**Erwin Gäbele**

Germany

**Gary Galante**

Canada

**Giovanni Galati**

Italy

**Francesca Galeazzi**

Italy

**Enrico Galmozzi**

Italy

**Shivanand Gamanagatti**

India

**Rita Garcia Martinez**

Spain

**Pramod Garg**

India

**Manuela Gariboldi**

Italy

**Zhongming Ge**

USA

**Krisztina Gecse**

Hungary

**Fernand-Pierre Gendron**

Canada

**Uday Ghoshal**

India

**Cinzia Giacometti**

Italy

**Catia Giovannini**

Italy

**Robert Gish**

USA

**Shannon Glaser**

USA

**Weihua Gong**

China

**Elisabetta Goni**

Italy

**Jeroen Goos**

Netherlands

**Melita Gordon**

UK

**Ramon Gorter**

Netherlands

**Gunnar Gothberg**

Sweden

**Peter Green**

USA

**Jennifer Griffin**

Canada

**Shannon Griffin**

USA

**Mehmet Haberal**

Turkey

**Luciana Haddad**

Brazil

**Chad Hall**

USA

**Barry Hall**

Ireland

**Heather Hampel**

USA

**Yuyan Han**

USA

**Kenichi Harada**

Japan

**Elaine Harkness**

UK

**Muhammad Hasan**

USA

**Cesare Hassan**

Italy

**Benjamin Heidrich**

Germany

**Fernando Herbella**

Brazil

**Tiberiu Hershcovici**

USA

**Toshifumi Hibi**

Japan

**Hatanaka Hisashi**

Japan

**Joerg Hoffmann**

Germany

**Christoph Höner Zu Siederdissen**

Germany

**Ping-Hsin Hsieh**

Taiwan

**Jan-Sing Hsieh**

Taiwan

**Chien-Ning Hsu**

Taiwan

**Pin-I Hsu**

Taiwan

**Qin Huang**

USA

**Patrick Hughes**

Australia

**Yee Tak Hui**

Hong Kong

**Hung-Hsu Hung**

Taiwan

**Richard Hunt**

Canada

**Christine M. Hunt**

USA

**Musharraf Husain**

India

**Jong Jin Hyun**

Korea, South

**Marietta Iacucci**

Canada

**Gianluca Ianiro**

Italy

**Sergio Iannazzo**

Italy

**Gasim Ibrahim**

Saudi Arabia

**Tsukasa Ikeura**

Japan

**Jun Imamura**

Japan

**Akira Imoto**

Japan

**Patrick Ingiliz**

Germany

**Sergejs Isajevs**

Latvia

**Claudio Isella**

Italy

**Genichiro Ishii**

Japan

**Jeremie Jacques**

France

**Khaled Jadallah**

Jordan

**Jana Jandova**

USA

**Julien Jarry**

France

**Irma Järvelä**

Finland

**Peter Jepsen**

Denmark

**Ran Jin**

USA

**Tatehiro Kagawa**

Japan

**Rakesh Kalapala**

India

**Frank Kallenberg**

Netherlands

**Julia Kälsch**

Germany

**Nobuhiko Kamada**

USA

**Tatsuo Kanda**

Japan

**Arne Kandulski**

Germany

**Kenitiro Kaneko**

Japan

**Eun-Suk Kang**

Korea, South

**Wei-Yu Kao**

Taiwan

**Konstantinos Karmiris**

Greece

**David Katzka**

USA

**Yasunari Kawabata**

Japan

**Robin Kelley**

USA

**Paul Kelly**

UK

**Kawada Kenro**

Japan

**Arif Ali Khan**

India

**Alexander Khoruts**

USA

**Jan Kielstein**

Germany

**Kyoung Kim**

Korea, South

**Ingorn Kimkong**

Thailand

**Jens Kittner**

Germany

**Anne Knudsen**

Denmark

**Masaaki Kobayashi**

Japan

**S Komatsu**

Japan

**Tine Kopp**

Denmark

**Uri Kopylov**

Canada

**Jay Kostman**

USA

**Anastasios Koulaouzidis**

UK

**Aleksander Krag**

Denmark

**Rajesh Krishnamoorthi**

USA

**Suneeta Krishnareddy**

USA

**Chikara Kunisaki**

Japan

**Chao-Hung Kuo**

Taiwan

**Juozas Kupcinskas**

Lithuania

**Vincenzo La Mura**

Italy

**Stephen Lagana**

USA

**Terry Lairmore**

USA

**Yan Lam**

Australia

**Laura Lamberti**

USA

**Dora Lam-Himlin**

USA

**Frank Lammert**

Germany

**Linda Law**

USA

**Cedric Le May**

France

**Hye-Seung Lee**

USA

**Tsung-Chun Lee**

Taiwan

**Jae-Hoon Lee**

Korea, South

**Teng-Yu Lee**

Taiwan

**Philippe Lehours**

France

**Maureen Leonard**

USA

**Maria Liz Leoz**

Spain

**Cosmas Rinaldi Lesmana**

Indonesia

**Cynthia Levy**

USA

**David Lewin**

USA

**Stephen Lewis**

UK

**James Lewis**

USA

**Yue Li**

China

**Chris Liacouras**

USA

**Wei-Chih Liao**

Taiwan

**Lev Lichtenstein**

Israel

**Paolo Limongelli**

Italy

**Lian-Jie Lin**

China

**Alexander Link**

Germany

**Jyh-Ming Liou**

Taiwan

**Tao Liu**

Australia

**Huwei Liu**

China

**Gin-Ho Lo**

Taiwan

**Wai-Kit Lo**

USA

**Josefa Leon Lopez**

Spain

**Vicente Lorenzo-Zúñiga García**

Spain

**Albert Lowenfels**

USA

**Ligong Lu**

China

**Yongke Lu**

USA

**Claudio Luchini**

Italy

**Jonas Ludvigsson**

Sweden

**Rashid Lui**

Hong Kong

**Jiing-Chyuan Luo**

Taiwan

**Stefan Lüth**

Germany

**Francesco Luzza**

Italy

**Benjamin Maasoumy**

Germany

**Laurence Macia**

Australia

**Giovanni Maconi**

Italy

**Kaushal Madan**

India

**Varocha Mahachai**

Thailand

**Avik Majumdar**

UK

**Govind Makharia**

India

**Richard Makins**

UK

**Mariano Malaguarnera**

Italy

**Shahid Malik**

USA

**Rosalie Mallant-Hent**

Netherlands

**Giuseppe Malleo**

Italy

**Andrew Mallett**

Australia

**Vivek Mangla**

India

**Murugu Manickam**

USA

**Hendrik Manner**

Germany

**Giovanni Marchegiani**

Italy

**Giulio Marchesini**

Italy

**Adil Mardinoglu**

Sweden

**Alberto Mariani**

Italy

**Monica Mars**

Netherlands

**Irene Martinucci**

Italy

**Riruke Maruyama**

Japan

**Hitoshi Maruyama**

Japan

**Marco Marzioni**

Italy

**Daiuske Masuda**

Japan

**Satohiro Masuda**

Japan

**Kei Matsueda**

Japan

**Dimitrios Mavridis**

Greece

**Jens Mayer**

Germany

**Steven Mcelroy**

USA

**Thomas Mcgarrity**

USA

**Cristina Meazza**

Italy

**Makoto Meguro**

Japan

**Arianeb Mehrabi**

Germany

**Fanyin Meng**

USA

**Shahin Merat**

Iran

**Ahmed Messallam**

USA

**Evangelos Messaris**

USA

**James Mezhir**

USA

**Pal Miheller**

Hungary

**Antonina Mikocka-Walus**

Australia

**Danilo Miskovic**

UK

**Benjamin Misselwitz**

Switzerland

**Tatsuya Miyazaki**

Japan

**Meir Mizrahi**

Israel

**Mansour Mohamadzadeh**

USA

**Paola Molteni**

Italy

**Giuseppe Montalto**

Italy

**Senador Moran Sanchez**

Spain

**F Motoi**

Japan

**Stefan Mueller-Lissner**

Germany

**Chris Mulder**

Netherlands

**Mohamed Mutalib**

UK

**Alessio Naccarati**

Italy

**Sellama Nadifi**

Morocco

**Kenji Nagahama**

Japan

**Hayato Nakagawa**

Japan

**Atsushi Nakajima**

Japan

**Masanao Nakamura**

Japan

**Jun Nakamura**

Japan

**Cindy Neuzillet**

France

**Lorenzo Nicolè**

Italy

**Abdelrahman Nimeri**

United Arab Emirates

**Madunil Niriella**

Sri Lanka

**Alireza Norouzi**

Iran, Islamic Republic Of

**Thiago Nunes**

Brazil

**Ichiro Oda**

Japan

**Takeshi Ogura**

Japan

**Yuki Ohya**

USA

**Shiro Oka**

Japan

**Hussein Okasha**

Egypt

**Kazuichi Okazaki**

Japan

**Monica Oleastro**

Portugal

**Nicholas Onaca**

USA

**David Ong**

Singapore

**Shoko Ono**

Japan

**Sofia Ornelas-Arroyo**

Mexico

**Benjamin Otto**

Germany

**Richard Owen**

UK

**Gokben Ozbey**

Turkey

**Fabio Pace**

Italy

**Amanda Page**

USA

**Sujoy Pal**

India

**Shanthi Palaniappan**

Malaysia

**Senthilnathan Palanisamy**

India

**Giovanni Pallabazzer**

Italy

**Vincenzo Palmieri**

USA

**Gopanandan Parthasarathy**

USA

**Robert Partyka**

Poland

**Eleonora Patsenker**

Switzerland

**Natalia Pedersen**

Denmark

**Gianmaria Pennelli**

Italy

**Luit Penninga**

Denmark

**Sharon Perrella**

Australia

**Raffaele Pezzilli**

Italy

**Phoebe Phillips**

Australia

**Guillaume Piessen**

France

**Alejandro Piscoya**

Peru

**Joseph Pisegna**

USA

**Salvador Pita-Fernández**

Spain

**Michael Pitiakoudis**

Greece

**Marco Pizzi**

Italy

**Lorenzo Polimeno**

Italy

**Kevin Pollock**

UK

**Hans-Christian Pommergaard**

Denmark

**Alina Popp**

Finland

**Dario Porett**

Italy

**Hasmukh Prajapati**

USA

**Jan Preiss**

Germany

**Timothy Price**

Australia

**Jesus Prieto**

Spain

**Chethan Puttarajappa**

Panama

**M. Masudur Rahman**

Bangladesh

**H Ramesh**

India

**Jens Rasenack**

Germany

**Norman Ratcliffe**

UK

**Matteo Ravaioli**

Italy

**Ratha Ray**

USA

**Pradeep Rebala**

India

**Helen Reeves**

UK

**Norelle Reilly**

USA

**Helen Remotti**

USA

**Johannes Rey**

Germany

**Silvia Ribback**

Germany

**Claudio Ricci**

Italy

**Thomas Ritz**

Germany

**Mar Riveiro-Barciela**

Spain

**Juan Carlos Roa**

Chile

**Ian Roberts-Thomson**

Australia

**Xavier Roblin**

France

**Michael Roerecke**

Canada

**Yishai Ron**

Israel

**Emanuele Rondonotti**

Italy

**Jonas Rosendahl**

Germany

**Christophe Rosty**

Australia

**Manolis Roulis**

USA

**Andrew Rowland**

Australia

**Enrica Rumiato**

Italy

**Salvatore Russo**

Italy

**Akiko Saka**

Japan

**Efstratios Saliakellis**

Greece

**Amanda Salis**

Australia

**Sundeep Saluja**

India

**Madhusudhan Sanaka**

USA

**Olof Sandström**

Sweden

**Angelo Sangiovanni**

Italy

**Roberto Santambrogio**

Italy

**Deborah Saraggi**

Italy

**Samantha Sarcognato**

Italy

**Giovanni Sarnelli**

Italy

**Stefan Sauerland**

Germany

**Jeremy Schaefer**

USA

**David Schaeffer**

Canada

**Christopher Schalchta**

Canada

**Joern M. Schattenberg**

Germany

**Annalisa Schiepatti**

Italy

**Robert Schierwagen**

Germany

**Margaret Schneider**

Canada

**Jonathan Schwartz**

USA

**Andrada Seicean**

Romania

**Neil Shah**

UK

**Muhammed Sherid**

USA

**Oren Shibolet**

Israel

**Yasuhiro Shirakawa**

Japan

**Elham Shirvani**

Germany

**Ivy Shiue**

UK

**Shailesh Shrikhande**

India

**Sadiq Sikora**

India

**Siddharth Singh**

USA

**Vikesh Singh**

USA

**Harkirat Singh**

USA

**Manish Singla**

USA

**Marianne Sinn**

Germany

**Venkatramani Sitaram**

India

**Mamata Sivagnanam**

USA

**Ali Kemal Sivrioglu**

Turkey

**Antonio Solins**

Italy

**Amornyotin Somchai**

Thailand

**Ming Song**

USA

**Bridget Southwell**

Australia

**Margarida Souto-Carneiro**

Portugal

**Ioan Sporea**

Romania

**Ankur Srivastava**

UK

**Amitabh Srivastava**

USA

**Peter Stanich**

USA

**Horia Stefanescu**

Romania

**Costin Teodor Streba**

Romania

**Venkat Subramanian**

UK

**Mitsushige Sugimoto**

Japan

**Xueying Sun**

New Zealand

**Kai-Feng Sung**

Taiwan

**Takuya Suzuki**

Japan

**Ingvar Syk**

Sweden

**Wing-Kin Syn**

UK

**Shun Fung Sze**

Hong Kong

**Sheryl Szeinbach**

USA

**Zoltan Szepes**

Hungary

**James Tabibian**

USA

**Frank Tacke**

Germany

**Wei-Chen Tai**

Taiwan

**Yoshitsugu Tajima**

Japan

**Masanobu Takahashi**

Japan

**Shao-Tao Tang**

China

**Elliot Tapper**

USA

**Giovanni Tarantino**

Italy

**Alex Tarr**

UK

**Daynijela Tasic**

Serbia

**Julia Tchou**

USA

**Sooraj Tejaswi**

USA

**Koji Teramoto**

Japan

**Traci Testerman**

USA

**Zhen Tian**

USA

**Hans Tillmann**

USA

**Kazutomo Togashi**

Japan

**Michele Tonerini**

Italy

**Francesco Tovoli**

Italy

**Caroline Trang**

France

**Jonel Trebicka**

Germany

**Guglielmo Trovato**

Italy

**Konstantinos Tsimogiannis**

Greece

**Yasuhiro Tsuda**

Japan

**Yishito Tsushima**

Japan

**Pamela Tuma**

USA

**Nesrin Ugras**

Turkey

**Holm Uhlig**

UK

**Shahid Ullah**

Australia

**Bella Ungar**

Israel

**Dino Vaira**

Italy

**Barbara Valdivia-Correa**

Mexico

**Leo Van Rossum**

Netherlands

**Niels Vande Casteele**

USA Minor Outlying Islands

**Sina Vatandoust**

Australia

**Nereo Vettoretto**

Italy

**Alessandro Vitale**

Italy

**Jiannis Vlachogiannakos**

Greece

**Jasper Vleugels**

Netherlands

**Pavel Vodicka**

Czech Republic

**Thomas Von Hahn**

Germany

**Ulrike Von-Arnim**

Germany

**Mihael Vucur**

Germany

**Veronika Vymetalkova**

Czech Republic

**Timothy Wade**

USA

**Mihir Wagh**

USA

**Homan Wai**

USA

**Maximilian Waldner**

Germany

**Liangjing Wang**

China

**Fusheng Wang**

China

**David Wang**

USA

**Chih-Yuan Wang**

Taiwan

**Jing-Houng Wang**

Taiwan

**David G Watt**

UK

**Malte Wehmeyer**

Germany

**Shu-Chen Wei**

Taiwan

**Martin Weltman**

Australia

**Thomas Wex**

Germany

**J. Patrick Whelan**

USA

**Edyta Wieczorek**

Poland

**Kathryn Williams**

Australia

**Christopher Williams**

USA

**Caroline Wilson**

UK

**Andrzej Wincewicz**

Poland

**Carl Wingren**

Sweden

**Jean Winter**

USA

**Piotr Witkowski**

USA

**Robert Wong**

USA

**Daniel Worthley**

Australia

**Dan Worthley**

Australia

**Alexander Wree**

USA

**Keng-Liang Wu**

Taiwan

**Yinglian Xiao**

China

**Javed Yakoob**

Pakistan

**Shih-Cheng Yang**

Taiwan

**Pw Yeh**

Taiwan

**Chih-Hsun Yi**

Taiwan

**Takehiko Yokobori**

Japan

**Xin Yongning**

China

**Ying Yuan**

China

**Arseniy Yuzhalin**

UK

**Shira Zelber-Sagi**

Israel

**Patrizia Zentilin**

Italy

**Lin Zhang**

China

**Qiang Zhang**

China

**Jingmin Zhao**

China

**Shusen Zheng**

China

**Lixin Zhu**

China

